# Aqua­bis(2,3-dimethyl-4-oxo-4*H*-pyrido[1,2-*a*]pyrimidin-9-olato)nickel(II)

**DOI:** 10.1107/S1600536809051563

**Published:** 2009-12-04

**Authors:** Huaihong Zhang, Yu Sun, Xiaodan Chen, Fei Yu, Zhihong Zou

**Affiliations:** a Ordered Matter Science Research Center, College of Chemistry and Chemical Engineering, Southeast University, Nanjing 210096, People’s Republic of China; b School of Chemistry and Biology, Yancheng Institute of Technology, Yancheng 224003, People’s Republic of China

## Abstract

In the crystal structure of the mononuclear title complex, [Ni(C_10_H_9_N_2_O_2_)_2_(H_2_O)], the Ni^II^ ion is five-coordinated in a distorted square-pyramidal geometry by two N atoms and two O atoms from 2,3-dimethyl-4-oxopyrido[1,2-*a*]pyrimidin-9-olate ligands and one O atom from a water mol­ecule. O—H⋯O hydrogen bonds between the coordinated water mol­ecule and the ligand connect adjacent mol­ecules, forming a ribbon parallel to the *b* axis.

## Related literature

For the design and synthesis of self-assembling systems with organic ligands containing N and O donors, see: Wei *et al.* (2009 ▶); Sun *et al.* (2008 ▶); Bayot *et al.* (2006 ▶); Chen *et al.* (2007 ▶). For structures of quinolin-8-ol complexes, see: Wu *et al.* (2006 ▶).
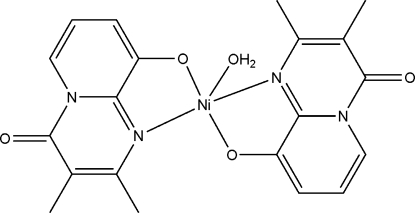


## Experimental

### 

#### Crystal data

[Ni(C_10_H_9_N_2_O_2_)_2_(H_2_O)]
*M* *_r_* = 455.11
Monoclinic, 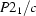

*a* = 13.4032 (14) Å
*b* = 12.1313 (12) Å
*c* = 12.4631 (11) Å
β = 99.386 (1)°
*V* = 1999.3 (3) Å^3^
*Z* = 4
Mo *K*α radiation
μ = 1.01 mm^−1^
*T* = 298 K
0.30 × 0.20 × 0.12 mm

#### Data collection

Rigaku SCXmini CCD area-detector diffractometer
Absorption correction: multi-scan (*CrystalClear*; Rigaku, 2005 ▶) *T*_min_ = 0.751, *T*_max_ = 0.888
10283 measured reflections
3520 independent reflections
2242 reflections with *I* > 2σ(*I*)
*R*_int_ = 0.045

#### Refinement

*R*[*F* ^2^ > 2σ(*F* ^2^)] = 0.049
*wR*(*F* ^2^) = 0.132
*S* = 1.03
3520 reflections
271 parameters
H-atom parameters constrained
Δρ_max_ = 0.41 e Å^−3^
Δρ_min_ = −0.41 e Å^−3^

### 

Data collection: *CrystalClear* (Rigaku, 2005 ▶); cell refinement: *CrystalClear*; data reduction: *CrystalClear*; program(s) used to solve structure: *SHELXS97* (Sheldrick, 2008 ▶); program(s) used to refine structure: *SHELXL97* (Sheldrick, 2008 ▶); molecular graphics: *SHELXTL* (Sheldrick, 2008 ▶); software used to prepare material for publication: *SHELXTL*.

## Supplementary Material

Crystal structure: contains datablocks I, New_Global_Publ_Block. DOI: 10.1107/S1600536809051563/zq2018sup1.cif

Structure factors: contains datablocks I. DOI: 10.1107/S1600536809051563/zq2018Isup2.hkl

Additional supplementary materials:  crystallographic information; 3D view; checkCIF report

## Figures and Tables

**Table 1:** Hydrogen-bond geometry (Å, °)

*D*—H⋯*A*	*D*—H	H⋯*A*	*D*⋯*A*	*D*—H⋯*A*
O5—H5*A*⋯O2^i^	0.85	1.83	2.672 (5)	173
O5—H5*B*⋯O4^ii^	0.85	1.85	2.669 (5)	162

Symmetry codes: (i) 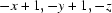; (ii) 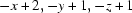.

**Fig. 1.**
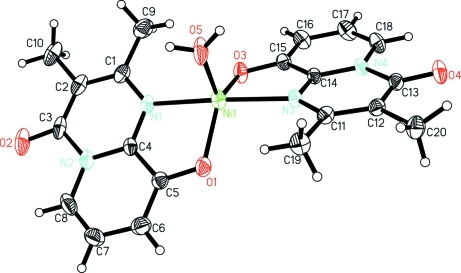
The molecular structure of the title molecule and the atom-numbering scheme. Displacement ellipsoids are drawn at the 30% probability level.

**Fig. 2.**
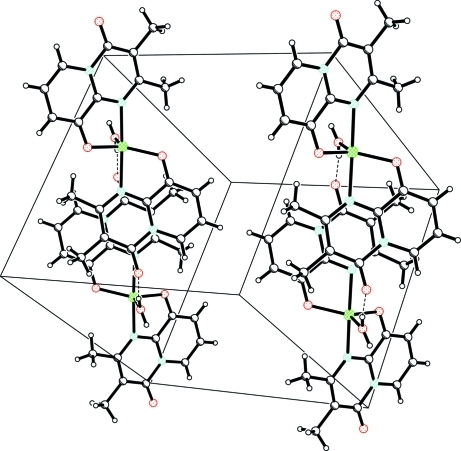
Crystal packing of compound (I). Hydrogen bonds are shown as dashed lines.

## supplementary crystallographic information

### Comment

Considerable attention has been paid to the design and synthesis of complexes with organic ligands containing N and O donors (Wei *et al.*, 2009; Sun *et al.*, 2008; Bayot *et al.*, 2006; Chen, *et al.*, 2007). Quinolin-8-ol is one such ligand and several crystal structures of complexes containing it have been reported (Wu *et al.*, 2006). We report here the synthesis and crystal structure of the title complex (I) (Fig. 1). In (I), the Ni atom is five-coordinated by two pyridine nitrogen atoms and two oxygen atoms from the hydroxy groups and one oxygen atom from a water molecule (Fig. 1). Intermolecular O—H···O hydrogen bonds (Fig.2 and Table 1) connecting the molecules of (I) define the crystal packing.

### Experimental

All chemicals used (reagent grade) were commercially available. An aqueous solution (5 ml) of Ni(Ac)_2_.4H_2_O (24.9 mg, 0.1 mmol) was added to an ethanol solution (10 ml) containing 2,3-dimethyl-9-hydroxylpyrido[1,2-*a*]pyrimidin-4-one (38.0 mg, 0.2 mmol) then filtered off. The resulting solution was continuously stirred for about 30 min and then filtered. The filtrate was slowly evaporated at room temperature over several days and colorless prism crystals suitable for X-ray analysis were obtained.

### Refinement

The H atoms bound to carbon were placed in geometrical positions and refined using a riding model, with C—H = 0.93Å and U_iso_(H) = 1.2Ueq(C) for aromatic H atoms, and with C—H = 0.96Å and U_iso_(H) = 1.5Ueq(C) for methyl H atoms. The H atoms of the coordinated water were located from the Fourier difference map and refined with a distance restraint of O–H = 0.85 Å.

### Crystal data

**Table:**

[Ni(C_10_H_9_N_2_O_2_)_2_(H_2_O)]	*F*(000) = 944
*M**_r_* = 455.11	*D*_x_ = 1.512 Mg m^−^^3^
Monoclinic, *P*2_1_/*c*	Mo *K*α radiation, λ = 0.71073 Å
Hall symbol: -P 2ybc	Cell parameters from 2294 reflections
*a* = 13.4032 (14) Å	θ = 2.3–23.7°
*b* = 12.1313 (12) Å	µ = 1.01 mm^−^^1^
*c* = 12.4631 (11) Å	*T* = 298 K
β = 99.386 (1)°	Prism, colorless
*V* = 1999.3 (3) Å^3^	0.30 × 0.20 × 0.12 mm
*Z* = 4	

### Data collection

**Table:**

Rigaku SCXmini CCD area-detector diffractometer	3520 independent reflections
Radiation source: fine-focus sealed tube	2242 reflections with *I* > 2σ(*I*)
graphite	*R*_int_ = 0.045
phi and ω scans	θ_max_ = 25.0°, θ_min_ = 1.5°
Absorption correction: multi-scan (*CrystalClear*; Rigaku, 200)	*h* = −15→15
*T*_min_ = 0.751, *T*_max_ = 0.888	*k* = −13→14
10283 measured reflections	*l* = −8→14

### Refinement

**Table:**

Refinement on *F*^2^	Primary atom site location: structure-invariant direct methods
Least-squares matrix: full	Secondary atom site location: difference Fourier map
*R*[*F*^2^ > 2σ(*F*^2^)] = 0.049	Hydrogen site location: inferred from neighbouring sites
*wR*(*F*^2^) = 0.132	H-atom parameters constrained
*S* = 1.03	*w* = 1/[σ^2^(*F*_o_^2^) + (0.0496*P*)^2^ + 2.7285*P*] where *P* = (*F*_o_^2^ + 2*F*_c_^2^)/3
3520 reflections	(Δ/σ)_max_ = 0.001
271 parameters	Δρ_max_ = 0.41 e Å^−^^3^
0 restraints	Δρ_min_ = −0.40 e Å^−^^3^

### Special details

**Table:**

Geometry. All e.s.d.'s (except the e.s.d. in the dihedral angle between two l.s. planes) are estimated using the full covariance matrix. The cell e.s.d.'s are taken into account individually in the estimation of e.s.d.'s in distances, angles and torsion angles; correlations between e.s.d.'s in cell parameters are only used when they are defined by crystal symmetry. An approximate (isotropic) treatment of cell e.s.d.'s is used for estimating e.s.d.'s involving l.s. planes.
Refinement. Refinement of *F*^2^ against ALL reflections. The weighted *R*-factor *wR* and goodness of fit *S* are based on *F*^2^, conventional *R*-factors *R* are based on *F*, with *F* set to zero for negative *F*^2^. The threshold expression of *F*^2^ > σ(*F*^2^) is used only for calculating *R*-factors(gt) *etc*. and is not relevant to the choice of reflections for refinement. *R*-factors based on *F*^2^ are statistically about twice as large as those based on *F*, and *R*- factors based on ALL data will be even larger.

### Fractional atomic coordinates and isotropic or equivalent isotropic displacement parameters (Å^2^)

**Table:**

	*x*	*y*	*z*	*U*_iso_*/*U*_eq_	
Ni1	0.79535 (4)	0.41721 (5)	0.19800 (5)	0.0428 (2)	
N1	0.6831 (2)	0.4337 (3)	0.0534 (3)	0.0351 (9)	
N2	0.5489 (3)	0.3281 (3)	−0.0425 (3)	0.0413 (10)	
N3	0.9220 (2)	0.3915 (3)	0.3261 (3)	0.0334 (8)	
N4	1.0960 (3)	0.4366 (3)	0.3602 (3)	0.0348 (9)	
O1	0.7277 (2)	0.2737 (3)	0.1993 (2)	0.0460 (8)	
O2	0.4536 (3)	0.3929 (3)	−0.1970 (3)	0.0690 (12)	
O3	0.9049 (2)	0.4701 (3)	0.1247 (2)	0.0460 (8)	
O4	1.1941 (2)	0.3960 (3)	0.5228 (3)	0.0536 (9)	
O5	0.7303 (2)	0.5312 (3)	0.2785 (3)	0.0679 (11)	
H5A	0.6697	0.5514	0.2562	0.082*	
H5B	0.7550	0.5674	0.3350	0.082*	
C1	0.6639 (3)	0.5152 (4)	−0.0221 (4)	0.0404 (11)	
C2	0.5870 (4)	0.5058 (4)	−0.1098 (4)	0.0475 (13)	
C3	0.5254 (3)	0.4119 (5)	−0.1233 (4)	0.0479 (13)	
C4	0.6274 (3)	0.3425 (4)	0.0416 (3)	0.0337 (10)	
C5	0.6514 (3)	0.2575 (4)	0.1225 (4)	0.0373 (11)	
C6	0.5915 (4)	0.1653 (4)	0.1126 (4)	0.0504 (13)	
H6	0.6050	0.1089	0.1634	0.061*	
C7	0.5104 (4)	0.1549 (5)	0.0275 (5)	0.0621 (15)	
H7	0.4700	0.0922	0.0231	0.075*	
C8	0.4899 (4)	0.2337 (5)	−0.0480 (5)	0.0586 (15)	
H8	0.4358	0.2252	−0.1045	0.070*	
C9	0.7298 (4)	0.6146 (4)	−0.0027 (4)	0.0582 (14)	
H9A	0.7661	0.6130	0.0703	0.087*	
H9B	0.6887	0.6798	−0.0130	0.087*	
H9C	0.7769	0.6149	−0.0530	0.087*	
C10	0.5654 (4)	0.5939 (5)	−0.1961 (4)	0.0653 (16)	
H10A	0.5696	0.6652	−0.1622	0.098*	
H10B	0.4987	0.5834	−0.2365	0.098*	
H10C	0.6142	0.5891	−0.2445	0.098*	
C11	0.9307 (3)	0.3468 (4)	0.4274 (3)	0.0352 (10)	
C12	1.0208 (3)	0.3451 (4)	0.4981 (3)	0.0362 (10)	
C13	1.1084 (3)	0.3907 (4)	0.4675 (4)	0.0378 (11)	
C14	1.0038 (3)	0.4335 (3)	0.2942 (3)	0.0311 (10)	
C15	0.9932 (3)	0.4783 (4)	0.1855 (3)	0.0360 (10)	
C16	1.0767 (4)	0.5258 (4)	0.1534 (4)	0.0466 (12)	
H16	1.0721	0.5558	0.0841	0.056*	
C17	1.1679 (4)	0.5290 (4)	0.2247 (4)	0.0527 (13)	
H17	1.2234	0.5623	0.2021	0.063*	
C18	1.1788 (3)	0.4855 (4)	0.3254 (4)	0.0480 (13)	
H18	1.2410	0.4883	0.3711	0.058*	
C19	0.8349 (3)	0.3004 (4)	0.4571 (4)	0.0484 (13)	
H19A	0.8131	0.3456	0.5121	0.073*	
H19B	0.8470	0.2268	0.4842	0.073*	
H19C	0.7833	0.2993	0.3938	0.073*	
C20	1.0318 (4)	0.2944 (4)	0.6096 (4)	0.0551 (14)	
H20A	0.9703	0.3043	0.6384	0.083*	
H20B	1.0866	0.3293	0.6566	0.083*	
H20C	1.0457	0.2170	0.6048	0.083*	

### Atomic displacement parameters (Å^2^)

**Table:**

	*U*^11^	*U*^22^	*U*^33^	*U*^12^	*U*^13^	*U*^23^
Ni1	0.0354 (3)	0.0546 (4)	0.0368 (4)	−0.0007 (3)	0.0008 (3)	0.0014 (3)
N1	0.0296 (19)	0.045 (2)	0.029 (2)	0.0054 (17)	0.0013 (15)	−0.0004 (17)
N2	0.034 (2)	0.052 (3)	0.035 (2)	0.0108 (19)	−0.0008 (17)	−0.0133 (18)
N3	0.0277 (19)	0.043 (2)	0.029 (2)	−0.0016 (16)	0.0040 (15)	0.0005 (16)
N4	0.0308 (19)	0.042 (2)	0.030 (2)	−0.0009 (16)	0.0009 (16)	−0.0070 (16)
O1	0.0438 (19)	0.053 (2)	0.0376 (19)	0.0011 (15)	−0.0052 (15)	0.0100 (15)
O2	0.051 (2)	0.105 (3)	0.044 (2)	0.028 (2)	−0.0141 (18)	−0.015 (2)
O3	0.0358 (18)	0.067 (2)	0.0329 (18)	−0.0040 (16)	−0.0014 (14)	0.0100 (16)
O4	0.042 (2)	0.075 (3)	0.038 (2)	0.0039 (17)	−0.0102 (16)	−0.0068 (16)
O5	0.048 (2)	0.098 (3)	0.048 (2)	0.028 (2)	−0.0204 (16)	−0.032 (2)
C1	0.042 (3)	0.046 (3)	0.035 (3)	0.012 (2)	0.011 (2)	0.007 (2)
C2	0.052 (3)	0.057 (3)	0.034 (3)	0.024 (3)	0.009 (2)	0.005 (2)
C3	0.037 (3)	0.072 (4)	0.032 (3)	0.025 (3)	−0.004 (2)	−0.010 (3)
C4	0.028 (2)	0.043 (3)	0.029 (2)	0.004 (2)	0.0021 (19)	−0.006 (2)
C5	0.034 (3)	0.039 (3)	0.040 (3)	0.003 (2)	0.008 (2)	−0.002 (2)
C6	0.049 (3)	0.042 (3)	0.062 (4)	0.002 (2)	0.014 (3)	0.003 (2)
C7	0.049 (3)	0.043 (3)	0.092 (5)	−0.005 (3)	0.005 (3)	−0.017 (3)
C8	0.037 (3)	0.066 (4)	0.068 (4)	−0.004 (3)	−0.006 (3)	−0.031 (3)
C9	0.062 (3)	0.052 (3)	0.062 (4)	0.005 (3)	0.013 (3)	0.017 (3)
C10	0.071 (4)	0.078 (4)	0.046 (3)	0.034 (3)	0.006 (3)	0.018 (3)
C11	0.040 (3)	0.038 (3)	0.028 (3)	0.003 (2)	0.008 (2)	0.002 (2)
C12	0.047 (3)	0.035 (3)	0.026 (2)	0.004 (2)	0.002 (2)	−0.0003 (19)
C13	0.041 (3)	0.038 (3)	0.030 (3)	0.006 (2)	−0.004 (2)	−0.008 (2)
C14	0.029 (2)	0.037 (2)	0.027 (2)	0.0029 (19)	0.0013 (18)	−0.0050 (18)
C15	0.037 (3)	0.042 (3)	0.028 (3)	−0.001 (2)	0.004 (2)	−0.004 (2)
C16	0.050 (3)	0.060 (3)	0.031 (3)	−0.011 (2)	0.010 (2)	0.000 (2)
C17	0.047 (3)	0.067 (4)	0.046 (3)	−0.014 (3)	0.015 (3)	−0.007 (3)
C18	0.029 (3)	0.067 (3)	0.047 (3)	−0.010 (2)	0.005 (2)	−0.008 (3)
C19	0.046 (3)	0.061 (3)	0.040 (3)	−0.004 (2)	0.012 (2)	0.004 (2)
C20	0.064 (3)	0.060 (3)	0.037 (3)	0.002 (3)	−0.004 (3)	0.007 (2)

### Geometric parameters (Å, °)

**Table:**

Ni1—O3	1.959 (3)	C6—H6	0.9300
Ni1—O1	1.964 (3)	C7—C8	1.338 (8)
Ni1—O5	1.991 (3)	C7—H7	0.9300
Ni1—N3	2.155 (3)	C8—H8	0.9300
Ni1—N1	2.160 (3)	C9—H9A	0.9600
N1—C4	1.329 (5)	C9—H9B	0.9600
N1—C1	1.360 (5)	C9—H9C	0.9600
N2—C4	1.370 (5)	C10—H10A	0.9600
N2—C8	1.387 (6)	C10—H10B	0.9600
N2—C3	1.429 (6)	C10—H10C	0.9600
N3—C14	1.327 (5)	C11—C12	1.374 (6)
N3—C11	1.361 (5)	C11—C19	1.503 (6)
N4—C14	1.368 (5)	C12—C13	1.406 (6)
N4—C18	1.389 (5)	C12—C20	1.505 (6)
N4—C13	1.433 (6)	C14—C15	1.445 (6)
O1—C5	1.297 (5)	C15—C16	1.374 (6)
O2—C3	1.239 (5)	C16—C17	1.390 (7)
O3—C15	1.301 (5)	C16—H16	0.9300
O4—C13	1.241 (5)	C17—C18	1.347 (7)
O5—H5A	0.8499	C17—H17	0.9300
O5—H5B	0.8499	C18—H18	0.9300
C1—C2	1.379 (6)	C19—H19A	0.9600
C1—C9	1.491 (7)	C19—H19B	0.9600
C2—C3	1.401 (7)	C19—H19C	0.9600
C2—C10	1.511 (7)	C20—H20A	0.9600
C4—C5	1.442 (6)	C20—H20B	0.9600
C5—C6	1.370 (6)	C20—H20C	0.9600
C6—C7	1.395 (7)		
			
O3—Ni1—O1	132.53 (14)	N2—C8—H8	119.8
O3—Ni1—O5	115.52 (16)	C1—C9—H9A	109.5
O1—Ni1—O5	111.75 (15)	C1—C9—H9B	109.5
O3—Ni1—N3	80.38 (12)	H9A—C9—H9B	109.5
O1—Ni1—N3	100.05 (13)	C1—C9—H9C	109.5
O5—Ni1—N3	94.82 (13)	H9A—C9—H9C	109.5
O3—Ni1—N1	93.07 (13)	H9B—C9—H9C	109.5
O1—Ni1—N1	80.18 (13)	C2—C10—H10A	109.5
O5—Ni1—N1	93.07 (13)	C2—C10—H10B	109.5
N3—Ni1—N1	171.37 (13)	H10A—C10—H10B	109.5
C4—N1—C1	119.3 (4)	C2—C10—H10C	109.5
C4—N1—Ni1	108.5 (3)	H10A—C10—H10C	109.5
C1—N1—Ni1	132.2 (3)	H10B—C10—H10C	109.5
C4—N2—C8	120.3 (4)	N3—C11—C12	122.3 (4)
C4—N2—C3	120.1 (4)	N3—C11—C19	115.5 (4)
C8—N2—C3	119.6 (4)	C12—C11—C19	122.2 (4)
C14—N3—C11	118.7 (3)	C11—C12—C13	120.4 (4)
C14—N3—Ni1	108.2 (3)	C11—C12—C20	122.8 (4)
C11—N3—Ni1	133.0 (3)	C13—C12—C20	116.8 (4)
C14—N4—C18	120.9 (4)	O4—C13—C12	127.5 (4)
C14—N4—C13	120.4 (4)	O4—C13—N4	117.0 (4)
C18—N4—C13	118.7 (4)	C12—C13—N4	115.5 (4)
C5—O1—Ni1	116.0 (3)	N3—C14—N4	122.7 (4)
C15—O3—Ni1	115.7 (3)	N3—C14—C15	117.7 (4)
Ni1—O5—H5A	120.6	N4—C14—C15	119.6 (4)
Ni1—O5—H5B	129.2	O3—C15—C16	124.7 (4)
H5A—O5—H5B	110.1	O3—C15—C14	117.3 (4)
N1—C1—C2	121.4 (4)	C16—C15—C14	118.0 (4)
N1—C1—C9	115.8 (4)	C15—C16—C17	119.9 (4)
C2—C1—C9	122.8 (4)	C15—C16—H16	120.0
C1—C2—C3	120.8 (4)	C17—C16—H16	120.0
C1—C2—C10	122.5 (5)	C18—C17—C16	122.4 (5)
C3—C2—C10	116.7 (5)	C18—C17—H17	118.8
O2—C3—C2	127.7 (5)	C16—C17—H17	118.8
O2—C3—N2	116.4 (5)	C17—C18—N4	119.1 (4)
C2—C3—N2	115.9 (4)	C17—C18—H18	120.4
N1—C4—N2	122.6 (4)	N4—C18—H18	120.4
N1—C4—C5	117.4 (4)	C11—C19—H19A	109.5
N2—C4—C5	120.0 (4)	C11—C19—H19B	109.5
O1—C5—C6	124.8 (4)	H19A—C19—H19B	109.5
O1—C5—C4	117.8 (4)	C11—C19—H19C	109.5
C6—C5—C4	117.4 (4)	H19A—C19—H19C	109.5
C5—C6—C7	121.0 (5)	H19B—C19—H19C	109.5
C5—C6—H6	119.5	C12—C20—H20A	109.5
C7—C6—H6	119.5	C12—C20—H20B	109.5
C8—C7—C6	121.0 (5)	H20A—C20—H20B	109.5
C8—C7—H7	119.5	C12—C20—H20C	109.5
C6—C7—H7	119.5	H20A—C20—H20C	109.5
C7—C8—N2	120.3 (5)	H20B—C20—H20C	109.5
C7—C8—H8	119.8		
			
O3—Ni1—N1—C4	133.7 (3)	N1—C4—C5—O1	−2.3 (6)
O1—Ni1—N1—C4	1.0 (3)	N2—C4—C5—O1	178.5 (4)
O5—Ni1—N1—C4	−110.5 (3)	N1—C4—C5—C6	177.6 (4)
O3—Ni1—N1—C1	−44.8 (4)	N2—C4—C5—C6	−1.6 (6)
O1—Ni1—N1—C1	−177.5 (4)	O1—C5—C6—C7	179.7 (4)
O5—Ni1—N1—C1	71.0 (4)	C4—C5—C6—C7	−0.1 (7)
O3—Ni1—N3—C14	6.0 (3)	C5—C6—C7—C8	1.1 (8)
O1—Ni1—N3—C14	137.8 (3)	C6—C7—C8—N2	−0.4 (8)
O5—Ni1—N3—C14	−109.1 (3)	C4—N2—C8—C7	−1.4 (7)
O3—Ni1—N3—C11	−176.6 (4)	C3—N2—C8—C7	−179.5 (5)
O1—Ni1—N3—C11	−44.9 (4)	C14—N3—C11—C12	1.0 (6)
O5—Ni1—N3—C11	68.3 (4)	Ni1—N3—C11—C12	−176.2 (3)
O3—Ni1—O1—C5	−87.5 (3)	C14—N3—C11—C19	−179.6 (4)
O5—Ni1—O1—C5	87.0 (3)	Ni1—N3—C11—C19	3.3 (6)
N3—Ni1—O1—C5	−173.6 (3)	N3—C11—C12—C13	0.2 (7)
N1—Ni1—O1—C5	−2.4 (3)	C19—C11—C12—C13	−179.2 (4)
O1—Ni1—O3—C15	−102.8 (3)	N3—C11—C12—C20	−179.0 (4)
O5—Ni1—O3—C15	82.8 (3)	C19—C11—C12—C20	1.7 (7)
N3—Ni1—O3—C15	−8.0 (3)	C11—C12—C13—O4	179.3 (4)
N1—Ni1—O3—C15	177.7 (3)	C20—C12—C13—O4	−1.6 (7)
C4—N1—C1—C2	0.9 (6)	C11—C12—C13—N4	−0.3 (6)
Ni1—N1—C1—C2	179.3 (3)	C20—C12—C13—N4	178.9 (4)
C4—N1—C1—C9	179.9 (4)	C14—N4—C13—O4	179.8 (4)
Ni1—N1—C1—C9	−1.7 (6)	C18—N4—C13—O4	−1.2 (6)
N1—C1—C2—C3	0.5 (7)	C14—N4—C13—C12	−0.6 (6)
C9—C1—C2—C3	−178.4 (4)	C18—N4—C13—C12	178.4 (4)
N1—C1—C2—C10	−178.7 (4)	C11—N3—C14—N4	−2.0 (6)
C9—C1—C2—C10	2.4 (7)	Ni1—N3—C14—N4	175.8 (3)
C1—C2—C3—O2	179.4 (4)	C11—N3—C14—C15	178.7 (4)
C10—C2—C3—O2	−1.4 (7)	Ni1—N3—C14—C15	−3.5 (4)
C1—C2—C3—N2	−0.9 (6)	C18—N4—C14—N3	−177.2 (4)
C10—C2—C3—N2	178.3 (4)	C13—N4—C14—N3	1.8 (6)
C4—N2—C3—O2	179.7 (4)	C18—N4—C14—C15	2.2 (6)
C8—N2—C3—O2	−2.1 (6)	C13—N4—C14—C15	−178.8 (4)
C4—N2—C3—C2	0.0 (6)	Ni1—O3—C15—C16	−171.8 (4)
C8—N2—C3—C2	178.2 (4)	Ni1—O3—C15—C14	8.5 (5)
C1—N1—C4—N2	−1.9 (6)	N3—C14—C15—O3	−2.9 (6)
Ni1—N1—C4—N2	179.4 (3)	N4—C14—C15—O3	177.8 (4)
C1—N1—C4—C5	179.0 (4)	N3—C14—C15—C16	177.5 (4)
Ni1—N1—C4—C5	0.3 (4)	N4—C14—C15—C16	−1.9 (6)
C8—N2—C4—N1	−176.8 (4)	O3—C15—C16—C17	−179.2 (4)
C3—N2—C4—N1	1.4 (6)	C14—C15—C16—C17	0.4 (7)
C8—N2—C4—C5	2.3 (6)	C15—C16—C17—C18	0.8 (8)
C3—N2—C4—C5	−179.5 (4)	C16—C17—C18—N4	−0.6 (8)
Ni1—O1—C5—C6	−176.6 (3)	C14—N4—C18—C17	−0.9 (7)
Ni1—O1—C5—C4	3.3 (5)	C13—N4—C18—C17	−179.9 (4)

### Hydrogen-bond geometry (Å, °)

**Table:**

*D*—H···*A*	*D*—H	H···*A*	*D*···*A*	*D*—H···*A*
O5—H5A···O2^i^	0.85	1.83	2.672 (5)	173
O5—H5B···O4^ii^	0.85	1.85	2.669 (5)	162

Symmetry codes: (i) −*x*+1, −*y*+1, −*z*; (ii) −*x*+2, −*y*+1, −*z*+1.
